# The Anesthetic Management of a Patient With Advanced Heart Failure Undergoing Non-cardiac Surgery

**DOI:** 10.7759/cureus.103869

**Published:** 2026-02-18

**Authors:** Renata G Falchetti, Ivandete C Pereira Pimentel, Luísa C Alves Ribeiro de Castro, Almir A Pimentel Jr

**Affiliations:** 1 Department of Anesthesiology, Instituto Maria Schmitt, Sombrio, BRA; 2 Surgery Center, Foundation of Oncology Control Center of the State of Amazonas, Manaus, BRA; 3 Department of Medicine, School od Medicine, Universidade do Sul de Santa Catarina (UNISUL), Tubarão, BRA

**Keywords:** brachial plexus block, heart failure, orthopedic surgery, permanent pacemaker, regional anestesia, ultrasound

## Abstract

Severe heart failure (HF) is associated with high perioperative morbidity and mortality during non-cardiac surgery. Anesthetic management for patients with critically reduced left ventricular ejection fraction (LVEF) requires strategies that minimize myocardial depression and autonomic instability. We present the case of a 73-year-old male with severe HF (LVEF 18%), a permanent pacemaker, severe anemia, and ongoing clopidogrel therapy who required urgent fixation of an open distal humerus fracture. General anesthesia was considered prohibitively high risk. An ultrasound-guided supraclavicular brachial plexus block was performed using 20 mL of 0.5% ropivacaine. Surgery proceeded uneventfully without hemodynamic instability, and the patient was discharged from the ICU after 24 hours.

Regional anesthesia provides significant hemodynamic advantages in high-risk cardiac patients. Ultrasound guidance allows for precise needle placement and may reduce complications, even in patients receiving antiplatelet therapy, when the benefits outweigh the risks. An ultrasound-guided supraclavicular block proved to be a safe and effective anesthetic strategy in a patient with severe HF undergoing emergency orthopedic surgery.

## Introduction

Severe heart failure (HF) represents the advanced and refractory stage of the disease, characterized by symptoms at rest or with minimal exertion, severe functional limitation, and frequently reduced left ventricular ejection fraction (LVEF <30-35%) [1,2]. At this stage, HF is associated with increased perioperative morbidity and mortality during non-cardiac surgery, resulting from impaired cardiac output and limited hemodynamic reserve. In these patients, the markedly reduced ability to augment cardiac output means that even modest changes in preload, afterload, or heart rate - including those induced by autonomic fluctuations - may precipitate significant hemodynamic instability or circulatory collapse. Consequently, individuals with severe HF are at high risk of hemodynamic decompensation, including hypotension and myocardial depression, during anesthetic and surgical procedures, particularly when exposed to general anesthesia [3].

Regional anesthesia, when feasible, is advantageous in urgent orthopedic procedures because it preserves spontaneous ventilation, minimizes autonomic fluctuations, and reduces exposure to myocardial depressant anesthetic agents [4]. However, the presence of antiplatelet therapy and cardiac implantable electronic devices (CIEDs) adds additional complexity to perioperative planning. This report describes the perioperative management of a patient with severe HF (LVEF 18%), anemia, a permanent pacemaker, and ongoing clopidogrel therapy, undergoing emergency correction of an open humeral fracture. At admission, the patient exhibited clinical features consistent with advanced HF, including markedly reduced functional capacity, dyspnea on minimal exertion, baseline hypotension, and signs of limited cardiac reserve, further increasing perioperative risk.

## Case presentation

A 73-year-old male (65 kg, 168 cm) with advanced HF (LVEF 18%) and a permanent pacemaker was admitted for urgent surgical fixation of an open distal humerus fracture sustained from a drone-related trauma. The patient had advanced HF characterized by dyspnea on minimal exertion, severe functional limitation, baseline hypotension, severe left ventricular systolic dysfunction, and pacemaker dependence, while receiving chronic pharmacological therapy. His chronic medications included metoprolol, furosemide, clopidogrel, prednisone, digoxin, amiodarone, dapagliflozin, sacubitril/valsartan, finasteride, doxazosin, and atorvastatin. Laboratory tests revealed a hemoglobin level of 6.8 g/dL and a hematocrit of 21%. Upon arrival in the operating room, monitoring showed a blood pressure of 58/38 mmHg, heart rate of 55 bpm, and SpO₂ of 88%. Oxygen was administered at 3 L/min, and a transfusion of one unit of packed red blood cells was initiated. After transfusing one unit, blood pressure stabilized at the patient’s baseline (approximately 90/58 mmHg) without the need for vasoactive support.

**Figure 1:**
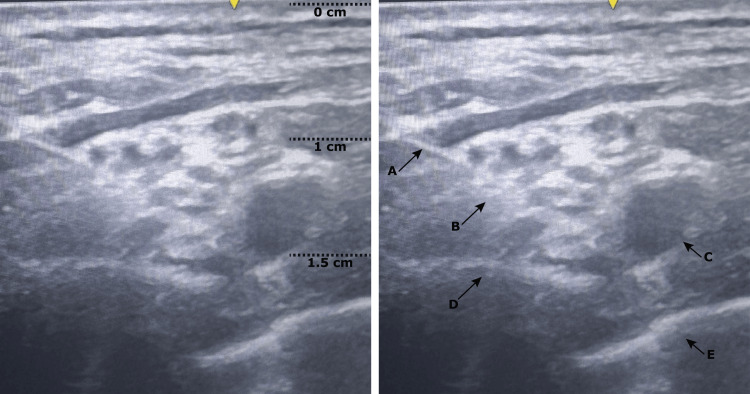
Ultrasound-guided supraclavicular brachial plexus block Transverse ultrasound image of the right supraclavicular region. The needle and relevant anatomical structures are identified by arrows and letters. The needle (A) is visualized advancing toward the brachial plexus (B), at an approximate depth of 1.5 cm, located lateral to the subclavian artery (C). The first rib (D) appears as a hyperechoic structure with posterior acoustic shadowing, and the pleura (E) is identified inferiorly. This visualization optimizes the needle trajectory while maintaining a safe distance from vascular and pleural structures

Given the extremely high risk associated with general anesthesia, the multidisciplinary team, including anesthesiology, orthopedics, and cardiology, opted for a regional technique. A supraclavicular brachial plexus block was performed under real-time ultrasound guidance (GE Healthcare). The patient was positioned supine, with the head turned contralaterally to the side of the block and the operative arm resting alongside the body. A high-frequency linear transducer was placed transversely in the supraclavicular fossa to identify the subclavian artery and, lateral to it, the brachial plexus. After adequate visualization of the plexus elements, a 50-mm Stimuplex® needle was advanced from lateral to medial using an in-plane technique, allowing continuous real-time visualization of the needle trajectory. The subclavian artery and first rib were used as anatomical safety landmarks to minimize the risk of vascular puncture and pleural injury (Figure 1).

A total of 20 mL of 0.5% ropivacaine (100 mg) was administered incrementally in 5-mL aliquots, with repeated aspiration before each injection. Adequate circumferential spread of the local anesthetic around the brachial plexus was observed at an approximate depth of 1.5 cm. Although a targeted injection into the “corner pocket” was not intentionally performed, satisfactory anesthetic distribution was achieved without complications.

The plexus block was performed without sedation because of concerns that the patient’s hypoxemia could worsen. The surgical procedure lasted 50 minutes and remained uneventful, with no clinically significant changes in blood pressure, heart rate, oxygen saturation, or respiratory rate observed during the incision or throughout the procedure. Intraoperative hemodynamic stability was defined as maintaining these parameters within acceptable limits, with no reductions greater than 20% from baseline and without the need for additional interventions such as sedation or vasopressors, and it was continuously monitored at five-minute intervals.

During surgery, intravenous dexamethasone (150 µg·kg⁻¹), ketoprofen (150 mg), dipyrone/metamizole (30 mg·kg⁻¹), ondansetron (100 µg·kg⁻¹), and 500 mL of balanced crystalloid solution (Plasma-Lyte®) were administered. At the end of the procedure, the patient presented with a blood pressure of 90×48 mmHg, heart rate of 55 bpm, and SpO₂ of 90%, and was transferred in stable condition to the ICU. After 24 hours, the patient was discharged to the ward with a blood pressure of 99×40 mmHg, peripheral oxygen saturation of 92%, heart rate of 55 bpm, respiratory rate of 15 breaths per minute, hematocrit of 24%, and hemoglobin level of 7.4 g/dL (post-transfusion).

## Discussion

Severe HF is associated with a markedly increased risk of perioperative morbidity, primarily due to impaired cardiac output and limited tolerance of acute hemodynamic changes [1,3]. An LVEF of 18%, as observed in this patient, reflects critical ventricular dysfunction and carries a high risk of perioperative decompensation. In patients with severe HF, reduced contractility, ventricular remodeling, and a pronounced dependence on preload and sympathetic tone substantially limit compensatory capacity. As a result, even small fluctuations in heart rate, preload, or afterload commonly seen during anesthetic and surgical stress can precipitate hypotension and hemodynamic collapse. This pathophysiologic vulnerability is emphasized in perioperative guidelines, which highlight the severely reduced physiologic reserve of these patients and the need for anesthetic strategies that minimize myocardial depression and autonomic instability [3].

Our patient was classified as Stage D heart failure according to American Heart Association (AHA) criteria, with a left ventricular ejection fraction of 18%. Although a formal functional assessment was not feasible due to the urgent presentation, his clinical profile was consistent with New York Heart Association (NYHA) functional class III-IV. The patient was also designated Physical Status IV E by the American Society of Anesthesiologists (ASA), reflecting severe systemic disease that poses a constant threat to life. Accordingly, current perioperative cardiovascular guidelines emphasize individualized planning for patients with advanced heart failure undergoing non-cardiac surgery, prioritizing strategies that preserve hemodynamic stability, minimize myocardial depression, and reduce autonomic stress [3]. In this context, anesthetic techniques that avoid abrupt changes in preload, afterload, and myocardial contractility are particularly important.

General anesthesia can exacerbate circulatory instability through myocardial depression, reductions in systemic vascular resistance, and sympathetic responses related to airway manipulation. When feasible, regional anesthesia may mitigate some of these effects and is therefore often considered for upper-limb procedures. However, the potential benefits are technique- and patient-specific and depend on avoiding extensive sympathectomy, respiratory compromise, or systemic local anesthetic toxicity. Current perioperative and regional anesthesia guidelines recommend an individualized risk-benefit assessment when considering regional techniques in patients with significant cardiovascular disease. Key principles include careful patient selection, preference for blocks with limited sympathetic effects, use of ultrasound guidance to enhance precision, and conservative dosing of local anesthetics [5].

In patients with advanced heart failure, reduced cardiac output and impaired hepatic perfusion may compromise the clearance of amide local anesthetics, increasing the risk of systemic toxicity. For this reason, using the lowest effective dose, incremental injection, and ultrasound guidance is recommended. Ropivacaine was chosen in this case because of its more favorable cardiotoxic profile compared with bupivacaine, and the total dose was kept at the minimum effective level in accordance with current safety guidelines [5]. Therefore, an ultrasound-guided supraclavicular brachial plexus block, with careful dose selection and incremental administration, aligned with recommendations from the American Society of Regional Anesthesia and Pain Medicine (ASRA) for the safe use of regional techniques in high-risk cardiac patients [5].

The presence of ongoing clopidogrel therapy added another challenge, given the increased bleeding risk associated with deep or intermediate-depth peripheral nerve blocks. ASRA and ESRA guidelines generally recommend discontinuing clopidogrel for five to seven days before performing neuraxial or deep peripheral nerve blocks [3,4,6,7]. However, in urgent surgical settings, these guidelines acknowledge that selected ultrasound-guided blocks may be considered when the anticipated benefits outweigh the risks and when meticulous technique is used [3,6-8]. The anesthetic strategy in this case followed the joint European Society of Anaesthesiology and Intensive Care and the European Society of Regional Anaesthesia( ESAIC/ESRA) recommendations on regional anesthesia in patients receiving antithrombotic therapy [9].

Although classified as an intermediate-depth block, the ultrasound-guided supraclavicular brachial plexus block was deemed appropriate in this specific clinical context. Compared with interscalene approaches, it may reduce the risk of phrenic nerve involvement and respiratory compromise, while allowing effective compression in the event of bleeding. Real-time ultrasound visualization of the pleura and subclavian vessels facilitates precise needle placement, potentially mitigating risks such as pneumothorax and intravascular injection (Figure 1) [9]. In patients with underlying cardiac disease, transfusion thresholds are often more permissive, with intervention considered when hemoglobin falls below 7-8 g/dL [10]. In this case, acute anemia was considered a contributing factor to baseline hypotension in the setting of severely reduced cardiac reserve. Correction of anemia was therefore included in the overall perioperative optimization strategy, aiming to improve myocardial oxygen delivery and support hemodynamic stability.

The presence of a permanent pacemaker required additional perioperative precautions because of the risk of electromagnetic interference from monopolar electrocautery. Current guidelines recommend identifying the device in advance, assessing pacing dependence, minimizing electromagnetic interference, and ensuring the availability of contingency measures such as transcutaneous pacing or external defibrillation when indicated [11]. In this case, bipolar electrocautery was used; the electrosurgical grounding pad was positioned away from the thorax, and emergency pacing capabilities were confirmed. No device reprogramming or magnet application was necessary. In this context, the stable perioperative course observed in this patient suggests that the adopted strategy, combining blood transfusion, supplemental oxygen, and ultrasound-guided regional anesthesia, was feasible and well-tolerated without immediate hemodynamic complications during this urgent surgical procedure. However, this observation is limited to a single case and should not be interpreted as evidence of broader perioperative benefit or general applicability.

## Conclusions

This report demonstrates that a carefully selected, ultrasound-guided peripheral nerve block may be feasible and was associated with a favorable perioperative course in a patient with advanced heart failure undergoing non-cardiac surgery. However, this represents an individual clinical experience, and the observed outcomes should not be generalized. Although the technique was well tolerated in this case, broader conclusions regarding safety and efficacy cannot be drawn. Further studies are needed to better define the role and optimal use of regional anesthesia techniques in this high-risk population.

## References

[1] McDonagh TA, Metra M, Adamo M (2021). 2021 ESC Guidelines for the diagnosis and treatment of acute and chronic heart failure. Eur Heart J.

[2] Kitai T, Kohsaka S, Kato T (2025). JCS/JHFS 2025 guideline on diagnosis and treatment of heart failure. Circ J.

[3] Thompson A, Fleischmann KE, Smilowitz NR (2024). 2024 AHA/ACC/ACS/ASNC/HRS/SCA/SCCT/SCMR/SVM Guideline for perioperative cardiovascular management for noncardiac surgery: A Report of the American College of Cardiology/American Heart Association Joint Committee on Clinical Practice Guidelines. Circulation.

[4] Martins LE, Ferraro LH, Takeda A, Munechika M, Tardelli MA (2017). Ultrasound-guided peripheral nerve blocks in anticoagulated patients - case series. Braz J Anesthesiol.

[5] Neal JM, Woodward CM, Harrison TK (2018). The American Society of Regional Anesthesia and Pain Medicine checklist for managing local anesthetic systemic toxicity: 2017 version. Reg Anesth Pain Med.

[6] Horlocker TT, Vandermeuelen E, Kopp SL, Gogarten W, Leffert LR, Benzon HT (2018). Regional anesthesia in the patient receiving antithrombotic or thrombolytic therapy: American Society of Regional Anesthesia and Pain Medicine evidence-based guidelines (fourth edition). Reg Anesth Pain Med.

[7] Douketis JD, Spyropoulos AC, Murad MH (2022). Perioperative management of antithrombotic therapy: an American College of Chest Physicians clinical practice guideline. Chest.

[8] Narouze S, Benzon HT, Provenzano D (2018). Interventional spine and pain procedures in patients on antiplatelet and anticoagulant medications (second edition): guidelines from the American Society of Regional Anesthesia and Pain Medicine, the European Society of Regional Anaesthesia and Pain Therapy, the American Academy of Pain Medicine, the International Neuromodulation Society, the North American Neuromodulation Society, and the World Institute of Pain. Reg Anesth Pain Med.

[9] Kietaibl S, Ferrandis R, Godier A (2022). Regional anaesthesia in patients on antithrombotic drugs: joint ESAIC/ESRA guidelines. Eur J Anaesthesiol.

[10] Carson JL, Brooks MM, Abbott JD (2016). Transfusion thresholds and other strategies for anemia in patients with cardiovascular disease. N Engl J Med.

[11] Shelton CL, Kearsley R (2022). How to write a case report in anaesthesia and peri-operative medicine. Anaesthesia.

